# Volcanic Ash as a Sustainable Binder Material: An Extensive Review

**DOI:** 10.3390/ma14051302

**Published:** 2021-03-08

**Authors:** Andrés Játiva, Evelyn Ruales, Miren Etxeberria

**Affiliations:** Department of Civil and Environmental Engineering, Universitat Politecnica de Catalunya-BarcelonaTECH, 08034 Barcelona, Spain; andres.jativa@upc.edu (A.J.); evelyn.ruales@upc.edu (E.R.)

**Keywords:** volcanic ash, treatments for reactivity, alkali-activated materials, curing method, strength properties, durability properties

## Abstract

The construction industry is affected by the constant growth in the populations of urban areas. The demand for cement production has an increasing environmental impact, and there are urgent demands for alternative sustainable solutions. Volcanic ash (VA) is an abundant low-cost material that, because of its chemical composition and amorphous atomic structure, has been considered as a suitable material to replace Portland cement clinker for use as a binder in cement production. In the last decade, there has been interest in using alkali-activated VA material as an alternative material to replace ordinary Portland cement. In this way, a valuable product may be derived from a currently under-utilized material. Additionally, alkali-activated VA-based materials may be suitable for building applications because of their good densification behaviour, mechanical properties and low porosity. This article describes the most relevant findings from researchers around the world on the role of the chemical composition and mineral contents of VA on reactivity during the alkali-activation reaction; the effect of synthesis factors, which include the concentration of the alkaline activator, the solution-to-binder ratio and the curing conditions, on the properties of alkali-activated VA-based materials; and the mechanical performance and durability properties of these materials.

## 1. Introduction

Ordinary Portland cement (OPC) is one of the most commonly used building materials for construction worldwide; it is also the most extensive manufactured product by mass on Earth. OPC is used in combination with water and aggregates and can also include chemical admixtures for the production of cement-based materials, such as concrete [1,2].

Currently, the global rise in urbanization has increased the need for cement. It is almost unimaginable that infrastructures could be developed without using OPC. OPC is inexpensive and adaptable for different types of constructions and requirements [3]. However, the production of one tonne of OPC generates 0.55 tonnes of carbon dioxide (CO_2_). An additional 0.39 tonnes of CO_2_ in fuel emissions are required for baking and grinding activities, which contribute to a total of 0.94 tonnes of CO_2_ [4,5]. Cement is the second most consumed material in the world, after water. The global demand for OPC is expected to increase by almost 200% by the year 2050 [6,7].

The scientific community is interested in achieving a better understanding of supplementary cementitious materials that could be used to partially or totally replace OPC. The major components of pozzolanic materials are silica (SiO_2_) and alumina (Al_2_O_3_), which do not have any hydraulic properties. However, when these components are mixed with lime or an alkaline product in the presence of water, they are capable of fixing calcium hydroxide, and they form stable compounds that have hydraulic cementitious properties [8,9]. Pozzolanic materials have been used since the Greek and Roman civilizations, which first used them in lime mortars to build such structures as bridges, basilicas, aqueducts, coliseums and temples; the use of these materials also became common at the beginning of the 20th century in the United States [10]. When pozzolanic materials are used as supplementary cementitious materials with OPC, several properties are improved with respect to 100% OPC [11,12]. For example, in the fresh state, cementitious materials improve the workability, reduce water retention/bleeding and lower the heat of hydration. In the hardened state, cementitious materials improve the resistance to sulphate and the alkali-aggregate reaction and increase the long-term strength.

Pozzolanic materials are commonly classified according to their origins as artificial or natural materials. The American Society for Testing and Material (ASTM) International classifies artificial pozzolanic material as Class F and Class C materials, which are focused on the different types of fly ash derived from the burning process of coal. Natural pozzolanic materials, or natural pozzolans (NPs), are classified as Class N materials. Class N materials include diatomaceous earth; opaline cherts and shales; calcined or uncalcined tuffs and volcanic ashes (VAs); and some clays and shales that require calcination to induce satisfactory properties.

Natural pozzolanic materials, such as VAs, can be used as supplementary cementitious materials. They require relatively few pre-treatments (i.e., grinding, calcination or chemical activation) before they can be used as alkali-activated materials (AAM) [13]. Although many natural resources undergo the clinkerization process at 1350–1450 °C to produce OPC, AAM materials are obtained at relatively low temperatures of 25–100 °C or 600–700 °C if the calcination process is required. Consequently, using natural pozzolanic materials to produce OPC results in a considerable reduction in CO_2_ emissions related to the production process [5]. In addition, natural pozzolanic materials are widely available in massive unexploited deposits around the world [12,14].

The most common VA used as a supplementary cementitious material is pyroclastic ash. Diagenetic processes can modify VA into zeolite-rich tuffs. The weathering process can cause zeolitisation or argillation of the ash, which can transform the pozzolan glass into zeolitic minerals or clay minerals. Although the zeolitisation of VA improves the pozzolanic properties, argillation reduces them [8].

The alkaline activation of VAs can be categorized into two groups. The first group can be characterized as alkali-activated binders without OPC and are known as geopolymers or inorganic alkaline polymers. The main reaction products in this group are a three-dimensional aluminosilicate network composed of sodium aluminosilicate hydrate (N-A-S-H) gel or potassium aluminosilicate hydrate (K-A-S-H) gel and zeolitic precipitates [15,16,17]. The second group, which is less studied and developed, is blended cement made of OPC and alkali-activated aluminosilicate binders. The blended cement is formed with a low proportion of OPC and a high proportion (i.e., over 70%) of VA, which is an aluminosilicate precursor. The main reaction products are very complex and comprised of cementitious gels. In highly alkaline media, such as 10 M NaOH (NH), there is a combination of sodium and calcium aluminosilicate hydrate (N-(C-)A-S-H) gels. In a slightly alkaline media, such as 2 M NH, the calcium silicate hydrate (C-S-H) product prevails. The reaction kinetics of this type of hybrid system alkaline activation are not well understood, particularly in the early stages [18,19,20].

The use of AAM in mortar or concrete production provides several advantages: there is an effective conversion of waste and by-products into supplementary cementitious materials; there is a reduction in greenhouse gas emissions during the production of concrete; and the concrete has better mechanical properties and durability compared with conventional concrete [12,20,21,22]. However, the production and application of AAM in large-scale use or industrial applications require further investigation. In addition, there is no consensus that alkali-activated cement has a better carbon footprint than that of OPC materials because of the dependence on the mix design, particularly the number of activators used. It has been reported that Na_2_SiO_3_ (NS) has a high carbon footprint, followed by NaOH [23,24].

To determine the full life-cycle assessment of AAM materials, the transportation of raw materials and the manufacturing process should be taken into account. However, few studies have provided this type of assessment. [2,25]. Thus, a great deal of research and development is required in this field.

The present report focusses on the use of NPs of volcanic origin for building and civil engineering applications. The results from studies on the use of VA as a supplementary cementitious material are described in the following sections. Specifically, papers published in recent years on the design and investigation of VA-based AAM materials are highlighted. The results of this review will inform scientific research about the methods that have the potential to be scaled up and contribute to real-world applications in this field.

## 2. Natural Volcanic Ashes 

**Figure 1:**
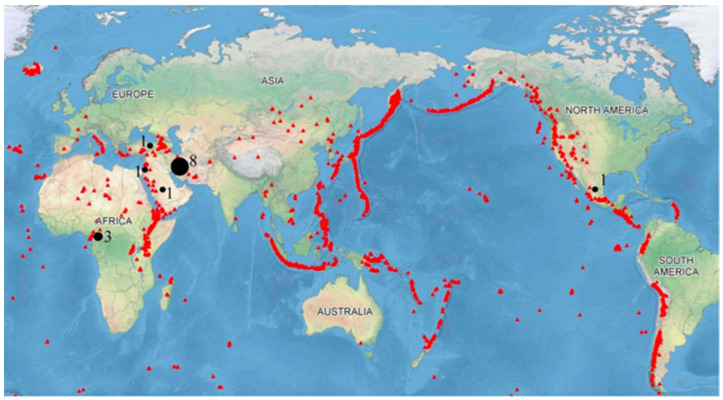
Global distribution of volcanoes (triangles) and countries conducting research on alkali-activated VA (circles). Adapted from [30]. Note: The numbers inside of map show the limited studies have been carried out, mainly in the Middle East.

VAs are NPs that are generated by magma fragmentation. The physical and chemical alteration processes involved in the formation of VAs affect their properties. For example, the leaching process or environmental conditions during the weathering process can modify the chemical and mineralogical properties of VAs [26]. VAs produced during a volcanic eruption consist of pyroclastic rocks, minerals and volcanic glass with diameters less than 2 mm [21]. Deposits of VA materials extend over approximately 0.84% of the world’s land surface or 124 million hectares, of which 60% are distributed in tropical regions [27]. These deposits are easily accessible, can be naturally mined and have a better cost–benefit ratio compared with the traditional quarrying method commonly used for clay mining. Additionally, in densely populated countries with high economic growth, these deposits can have a significant commercial value for the cement industry because the VA can be used in the production of blended cement [28]. Volcanic materials are obtained from Andisols exclusively found in regions with past or present volcanism, as shown in Figure 1 [12,26,29,30].

### 2.1. Properties of Volcanic Ash

#### 2.1.1. Chemical and Mineral Composition

Table 1 summarizes the chemical composition of VAs from different studies. The most essential and critical components of VAs are silica (SiO_2_) and alumina (Al_2_O_3_). While SiO_2_ is the main component of VA, Al_2_O_3_ is an essential component in most of the described pozzolans. VAs can also contain minor proportions of other pozzolanic oxides, such as ferric oxide (Fe_2_O_3_) and magnesium oxide (MgO), which are commonly found in acidic rock. Some of the VAs described in Table 1 contain 5% to 10% calcium oxide (CaO), which is responsible for the hydraulic cementitious properties of the material.

Some vitreous forms of VAs have a high loss of ignition (LOI), which indicates the presence of carbon dioxide (CO_2_) and water from the presence of carbonates and clay minerals, respectively [31,32,33,34].

The mineral content of VAs depends on the composition of the magma and the eruption conditions. VAs can vary from vitreous or amorphous to completely glassy or crystalline materials [37]. The most common minerals found in VAs are silicate minerals. There are different groups of silicate minerals: feldspars, such as albite and anorthite; the amphibole group, which includes tremolite; and the pyroxene group, which includes augite and diopside and less common quartzes [22,27,32,36,38,41,47]. Over the years, the weathering of volcanic glass produces minerals such as muscovite and biotite. These minerals belong to the mica group and montmorillonite clay, which are non-magnetic minerals [19,60].

#### 2.1.2. Physical Properties

The physical properties of VAs reported by different researchers are given in Table 2. The specific surface area (SSA) of VA, as determined using the Blaine method according to the ASTM C204 specifications, ranges from 240 to 1640 m^2^/kg, which is generally finer than the OPC SSA of 367 m^2^/kg [56]. The fineness of VA particles can be modified by removing the sandy fraction before blending with cement or by grinding the VA to a finer particle size [61]. Smaller VA particles have a more porous microstructure, and thus have a higher SSA and are more active in an alkaline medium [32].

Several researchers [56,61,62] reported that the bulk densities of different VAs range from 1649 to 2780 kg/m^3^. These values satisfy most of the criteria for Class N raw or calcined NPs according to ASTM C618 and are comparatively lower than the OPC bulk density of 3150 kg/m^3^ (determined following ASTM C188).

### 2.2. Standard Requirements and Test Procedures for Determination of VA Reactivity

According to the specifications of ASTM C618, the most critical chemical requirements for Class N pozzolanic materials for use in concrete are as follows: SiO_2_ + Al_2_O_3_ + Fe_2_O_3_ ≥ 70.0% and SO_3_ ≤ 4.0%; the moisture content should be ≤3.0%; and the LOI should be ≤ 10%. With respect to the fineness of the VA particles, the optimal grain size for Class N pozzolans should be ≤75 µm or the particles should be able to pass through a No. 200 sieve [11]. In accordance with the mechanical properties, the European Standard EN 450 and ASTM C618 specifications describe that the minimum strength activity index (SAI) of mortar samples produced with 25% or 20% of VA material, respectively, should be 75% of the compressive strength achieved by a 100% OPC control sample at 28 days [63].

The reactivity of a pozzolanic material depends significantly on several characteristics: the quality and quantity of the active phases; the SiO_2_/Al_2_O_3_ ratio; the mineral composition; and the duration of the curing process. In addition, the reaction rate depends on the SSA of the pozzolanic material; the water/solid material ratio; the alkaline content and the curing process (temperature and its duration) of the mixture [8,21].

The SiO_2_ and Al_2_O_3_ content, which typically makes up >70% of the VA mass, is responsible for reacting with the hydroxides of the OPC. The solubility of the glass phase of the VA increases as the pH of the solution increases; this affects the availability of the VA to react with calcium hydroxide (Ca(OH)_2_) and produces C-S-H products that increase the strength of the cementitious material. During the early stage, the reaction of the VA material mainly depends on the SSA, while the long-term activity depends on the chemical and mineralogical compositions of the VA material [50,64,65].

To assess the reaction rate of VA, the SAI of mortars and concretes made with a mixture of OPC and VA must first be determined. The reduction in free Ca(OH)_2_ in the hardened pozzolanic cement [66] can then be determined using X-ray diffraction (XRD), thermogravimetric analysis (TGA) or classical chemical titration analysis [40,48,49,67].

Several researchers [40,48,51] determined the SAI of different samples in order to characterize the pozzolanic activity of VAs. For example, Labbaci et al. [48] determined the SAI value of paste mixtures that used six types of VAs from basalt, olivine andesite, amphibole-biotite andesite, amphibole andesite, rhyodacite and scoria. The SAIs of all the mixtures ranged approximately from 82.11% to 91.91% that of the SAIs obtained using conventional mortar at 28 days. These results suggested that the pozzolanic activities of VAs, as measured by the SAI, were directly proportional with an increase in the active vitreous phases, which contain a higher SiO_2_ and Al_2_O_3_ content. Conversely, an increase in the Fe_2_O_3_ and MgO content of VAs adversely affected their pozzolanic activity. Hossain [40] performed an SAI test using 0–40% of VA material substituted for OPC. The determined SAI values at 7 days and 28 days were 78–100% and 67–100% of the compressive strength value, respectively, obtained using a 100% conventional mixture of OPC. The SAI values of mixtures produced using 10%, 20% and 30% of VA material had a higher compressive strength than the 75% of the strength value by conventional mixture (100% OPC), as required by ASTM C618. However, in general, the compressive strength of VA-based mortars generally decreased as the VA content increased.

There are two chemical test procedures that are used to determine the pozzolanic activity of VAs: the Frattini test and the saturated lime consumption test [68]. According to the EN 196-5 specification, the Frattini test involves a chemical titration to determine the dissolved Ca^2+^ and OH^−^ concentrations in a solution containing OPC and the pozzolanic materials. The saturated lime consumption test also measures the amount of calcium consumption when the pozzolanic material is mixed with a saturated lime (i.e., slaked lime or Ca(OH)_2_) solution instead of a solution of OPC and water. Hamidi et al. [49] analysed the pozzolanic activity using both chemical tests, and thus guaranteed their applicability.

TGA has been also used to determine the pozzolanic activity of VAs. Several studies, [49,59,67,69] used TGA to determine the amount of consumed Ca(OH)_2_, which was related to the pozzolanic reactivity. For example, Moropoulou et al. [59] found that the consumption rate of Ca(OH)_2_ in a mixture containing a lime/NP ratio of 1:3 was about 30% and 60% at 7 days and 28 days of curing, respectively. Askarinejad et al. [67] used the same method to analyse the pozzolan reactivity and determined that a mixture of lime/nanofabricated VA ratio of 1:1 had the highest Ca(OH)_2_ consumption rate of 26.83% at 9 days of curing.

The leaching test is a rapid chemical test that is used to determine the qualitative and quantitative reactive components of VA material [39,44]. The VA material is tested in different concentrations of NH or KH solutions at various temperatures. The leached components are analysed using inductively coupled plasma optical emission spectroscopy to determine the soluble fraction (i.e., amorphous phase) of the VA. For example, Djobo et al. [39] concluded that VA mixed in 12 M NH at 60 °C leached a maximum 24% dissolved Si and 21.5% dissolved Al with respect to the initial composition. Consequently, they demonstrated that VA material is less reactive in an alkaline solution than other aluminosilicates, such as fly ash and metakaolin (MK).

It is common to use more than one test method to determine the pozzolanic activity. In general, one of the test methods is qualitative and shows a trend of Ca(OH)_2_ consumption with time. When comparing different test methods, the temperature and the time of curing prior to testing should be considered.

## 3. Treatments to Increase the Reactivity of Natural Volcanic Ashes

This section may be divided by subheadings. It should provide a concise and precise description of the experimental results, their interpretation, and the experimental conclusions that can be drawn.

An increase in the pozzolanic activity of VA can overcome the important disadvantage of low early strength gains in cement containing VA materials [66,70]. Thus, different pre-treatments have been used to increase the potential reactivity of VAs [43,49,50,51,71,72]. There are three types of pre-treatment: mechanical activation (MA), the thermal method via calcination and chemical activation methods. MA exposes all the minerals in the VA to structural alterations, while the thermal method via calcination predominantly affects the clay minerals in the VA [73]. The chemical activation treatment method has become important in recent years in the synthesis of alkaline activated cements (AACs) because it is the only source of the reactive aluminosilicate precursor. Special attention will be paid to this method in the Section 4.

### 3.1. Mechanical Activation

MA is a grinding process that reduces the particle sizes of the VA material. This process increases the surface area of the material and exposes imperfections and active centres to a reaction. Thus, it increases the reactivity of the pozzolanic material. Because the active centres have a higher energy state than the normal structure, increasing the number of active centres enhances the reactivity of the VA material [66]. Furthermore, the grinding process induces the loss of crystallinity of the material and increases the amorphization of minerals. Consequently, it results in a high reactivity of minerals due to the breakage of valence bonds and the crystal structure distortions that lead to a partial or complete amorphization of the ground substance [74].

In their natural form, VA materials are composed of particles with diameters < 2 mm; some particles may even have a lower diameter than 1 μm [21]. However, in accordance with ASTM C618, approximately 2/3 of the VA particles must be <45 μm in order to qualify as a binder material in concrete production.

In several studies [38,50,75,76,77], the VA materials were milled before they were used as supplementary cementitious materials. These studies highlighted that finer sizes of VA material formed higher amounts of C-S-H and C-A-S-H gel phases, which are responsible for the achievement of the initial compressive strength in cement paste. In addition, smaller VA particles produced a denser pore structure, which had a greater compressive strength. Ardoğa et al. [77] also reported that blended cements that contained finer sizes of VAs and had a higher hydration heat due to the nucleation effect.

However, Djobo et al. [38] concluded that an extended VA milling process time could cause a reduction in the reactivity of VAs. The properties of VA materials are given in Table 1 and Table 2. The MA milling process was carried out for 30, 60, 90 and 120 min using a vibratory mill. After 30 min of milling, there was no relevant change in the reactivity capacity of VA. After 60 min of milling, structural changes occurred in the VA material. However, an important change in the degree of crystallinity of the VA was observed after 90 min of milling. XRD analysis showed that the degree of crystallinity decreased from 60% to 38%. This reduction in crystallinity was due to the increase in amorphization with milling. After 120 min of milling, the degree of crystallinity increased significantly and reached 60%. This phenomenon occurred due to the partial recrystallization of the glassy phase related to quartz formation. The degree of crystallinity (*Xc*) was determined to assess mechanical activation’s efectiveness in the crystallinity of volcanic ash. It is defined as the ratio of crystalline peaks’ area (*Ac*) to the sum of the amorphous phase area (*Aa*) and crystalline peaks’ area (*Ac*). The area of the crystalline peaks (*Ac*) was calculated as the integral of the upper zone of the smooth curve which connected peaks’ baselines, whereas the amorphous area (*Aa*) was obtained as the integral of the lower zone between the smooth curve and the linear baseline which connected the two points at 22° and 37° (2*θ*).

**Figure 2:**
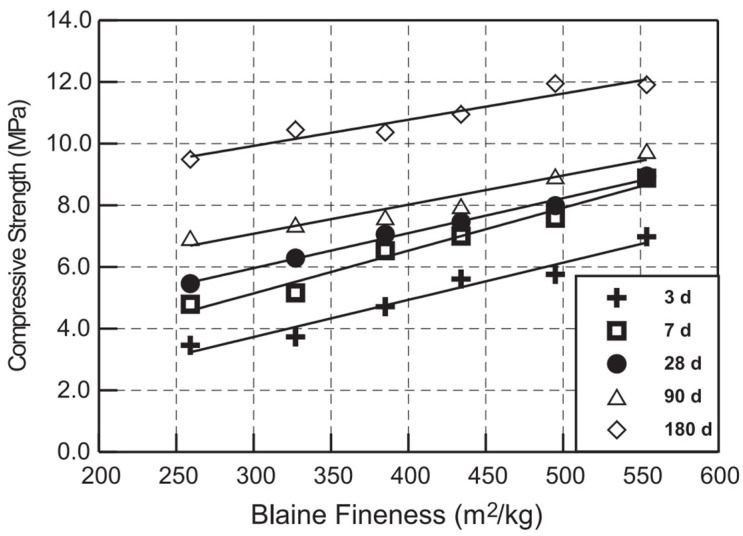
Relationship between the compressive strength of lime–natural pozzolan (NP) pastes and the Blaine fineness values of the NP [75].

The research work by Shi and Day [75] proved that prolonged grinding of VA material produced different Blaine fineness values of 259–554 m^2^/kg. In addition, the compressive strength of mixtures that were 20 wt.% hydrated lime and 80 wt.% pozzolan increased proportionally when the VA particles had a higher Blaine fineness value (see Figure 2). Kunal et al. [50] found that the cement paste produced using VA particles with a high fineness produced a higher amount of C-S-H and C-A-S-H gel phases than cement paste produced using coarser particles.

Kupwade-Patil et al. [76] produced cement pastes using 50% OPC and 50% of two types of milled VA materials. One type of VA material had particles with an average size of 6 μm; the other VA material had particles that were 17 μm. The samples made with the 6 μm VA particles had a compressive strength that was 15% higher than the samples made with the 17 μm VA particles. However, the samples made with the 6 μm VA particles had a 4% increase in embodied energy values compared to the samples made with the 17 μm VA particles due to the high energy used for milling. In addition, while the embodied energy value of the concrete made with the 6 μm VA particles was 17% less than the value of conventional OPC, there was also a 30% reduction in the compressive strength of the material compared with the conventional material. Therefore, the energy-efficient VA–OPC concrete mixtures could be engineered to have the required compressive strength for specific structural and non-structural applications.

### 3.2. Thermal Activation Via Calcination

The thermal activation via the calcination of aluminosilicate materials causes a loss of volatile components, as well as a change in entropy, a reorganization of atomic structures and a breakdown of the crystalline phases; these changes cause the VA materials to become amorphous and reactive [78]. Research conducted by Shi [66] showed that, at high temperatures, the calcination or preheating processes increased the density of VAs.

In general, when the amorphous components are at ≤40%, the VA material could be soluble in an alkaline environment [37,47]. In addition, when the semi-crystalline nature of the material is predominant, the VA has a deficiency in CaO and reactive Al_2_O_3_ components. Consequently, in order to increase the reactivity of alkali-activated binders and produced cementitious construction materials with a higher mechanical properties, it is common to use a calcination process > 700 °C [8,49,72,79]. Bondar et al. [43] concluded that calcination processes at 700, 800 and 900 °C positively influenced the mechanical properties of geopolymer pastes. The physical and chemical properties of the VA materials are given in Table 1 and Table 2, respectively. The raw VA–type 1 material was composed of a high percentage of zeolitic minerals: 40% clinoptilolite, 14% albite, 11% calcite and 32% quartz. The microstructure of the VA material changed to 26% mordenite and 38% opal after a calcination process at 700 and 800 °C, respectively. The calcination process modified the VA to be very reactive in alkaline solutions. In addition, the mixture produced with this VA material had a high compressive strength of 68.5 MPa when the samples were cured at 20 °C. The reactivity of the VA–type 2 material, which did not contain amorphous phases and had little soluble silica, increased after a calcination process at 900 °C. The VA–type 3 and VA–type 4 materials contained 33% and 25% amorphous phases, respectively, and more altered minerals, such as montmorillonite clay minerals; after the calcination process, the reactivity of the VA–type 3 material did not improve, although the VA–type 4 material achieved a slight activation capacity when it was treated at 800 °C. In order to determine the amorphous phases in VA materials, a rapid chemical was carried out. 200 mL of boiling 0.5 M NaOH was poured into a beaker containing 0.15 g of dry VA, and the resulting suspension was brought back to boiling as quickly as possible. After boiling for different times, the suspension was cooled quickly and filtered. The residue was washed with cold water and the residue dried at 105 °C overnight. The weight of undissolved material was determined by reweighing. The percentage of material dissolved was calculated and called alkali-solubility. The obtained solution after dissolution in NaOH allowed the determination of the amount of chemical elements, such as Si, Al, Fe, Mg and Ca, obtained by Inductively Coupled Plasma (ICP).

The values obtained by Kılıc and Sertabipoglu [51] corroborated that the pozzolanic activity of volcanic pumice material increased after a calcination process at 1000 °C. Mortar produced with 20% raw volcanic pumice material substituted for OPC achieved 73% of the SAI value obtained using conventional mortars produced with 100% OPC. However, the mortar produced with the calcined volcanic pumice achieved 107% of the SAI values obtained by the conventional mortar. Kani and Allahverdi [52] found that the SSA of the VA materials could change during calcination. However, they did not observe a correlation between the SSA, the calcination process and reactivity.

The mineral composition of the VA material can influence the effectiveness of the calcination process. According to Hamidi et al. and Askarinejad et al. [49,67], thermally treated andesite VA material after a calcination process at 800 and 700 °C had faster calcium fixation when added to a Ca(OH)_2_ solution than andesite VA material calcined at 900 °C. Additionally, VA materials with analcite and phillipsite as the main mineral phases showed a reduction in pozzolanic activity after the calcination process at 500–800 °C. Analcite kept its original crystal structure even after the loss of water. The phillipsite mineral transformed into metaphillipsite, which is a highly reactive product, after calcination to 500 °C, although calcination to 800 °C transformed it into a stable phase of feldspar with low pozzolanic activity [71,80]. Robayo-Salazar et al. [42] employed a calcination process at 700 °C with a Colombian VA material that was rich in montmorillonite, had a very high water-adsorption capacity and low reactivity. The calcination process was necessary to reduce the water demand and increase the reactivity of the material for use in the preparation of alkali-activated cements.

Some researchers [79,81,82] reached opposite conclusions with respect to the effect of the calcination process on the properties of VA materials. On one hand, the increase in the pozzolanic activity of vitreous, zeolitic and clay phases is due to calcination. On the other hand, a deactivation could occur due to the increase in the specific gravity of the VA materials when the calcination temperature is increased. The researchers also observed a reduction in porosity and an increase in crystallinity as the materials sintered at high temperatures.

## 4. Alkaline Activation of Natural Volcanic Ashes

VA material composed of aluminosilicate solids can develop appropriate binding properties after being exposed to alkaline conditions, which are induced by an alkaline activator with moderate to high alkalinity. The alkali-activated binders produced are also known as geopolymers [83].

Research into the synthesis of VA geopolymers has increased in recent years. The reaction mechanism starts with the OH^−^ ions from the alkaline activator breaking the siloxane (Si–O–Si) bonds and producing silanol (–Si–OH) and sialate (–Si–O^−^) species. The presence of the alkali cations normalizes the negative charge, while the formation of complex species, such as Si–O^−^–Na^+^ and Si–O–Al, hinder the reversion to siloxane [19]. In general, VA particles with a high fineness [84] and an alkaline activator solution with a pH of 12–14 [85] stimulate the dissolution of the components and the formation of monomeric aluminate and silicate units. The monomers clump together to form dimers and then oligomers, and continue to reorganize by increasing the gel system through the formation of three-dimensional polymeric chain and ring structures of aluminosilicate gels with Si–O–Al–O bonds. The reaction processes continue for quite some time, and after setting and prolonged curing under controlled hydrothermal conditions, zeolitic crystallites will grow [18,86,87].

**Figure 3:**
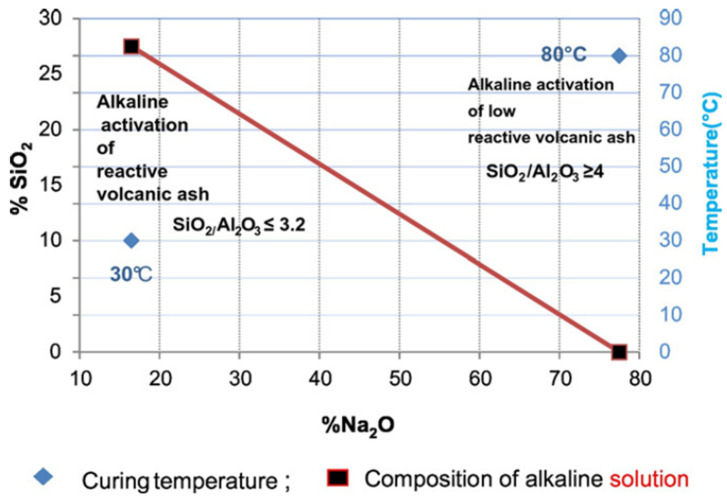
The optimal conditions investigated by Ndjock et al. [31] for the alkaline activation of VAs, taking into account the composition and content of the amorphous phases of VAs, the composition of the alkaline solution (SiO_2_ and Al_2_O_3_) and the curing temperature.

Several studies [31,37,38] have shown that alkali-activated VA materials have low reactivity. For example, Ndjock et al. [31] observed the effect of the composition and content of the amorphous phases of five samples of VA on reactivity. They concluded that the VA material could be suitable for alkaline activation depending on its amorphous phase content and SiO_2_/Al_2_O_3_ ratio. The VA material was classified as suitable for geopolymer synthesis when the SiO_2_/Al_2_O_3_ ratio was ≤3.9 and suitable for filler when the SiO_2_/Al_2_O_3_ ratio was >3.9. The optimal alkaline activation of VA material with a low amount of amorphous phase, a high SiO_2_/Al_2_O_3_ molar ratio and low reactivity was only possible using NaOH that contained 77.5 wt.% of Na_2_O and a curing temperature of 80 °C (refer to Figure 3). Some researchers [87,88] found that the optimum value of the SiO_2_/Al_2_O_3_ molar ratio for AAM varies from 3.3 to 4.5. The range depends on the Si and Al content available in the starting raw materials. Other researchers [32,33] suggested that the amorphous content must be at least 36% to obtain alkali-activated binders that have adequate properties.

The most common alkaline activators used for VA activations are mixtures of NaOH and KOH with sodium waterglass (nSiO_2_Na_2_O) or potassium waterglass (nSiO_2_K_2_O) [17,86]. These activators supply alkali-metal cations, increase the pH of the reaction mixture and accelerate the dissolution of the solid precursor, which promote strength development of the AAM [89]. Furthermore, the alkaline solution not only dissolves the alumina and silica precursors, but it also hydrolyses the surfaces of the particles, which allow reactions between the dissolved silicate and aluminate components and the particle surface to occur. In many cases, a surface reaction is responsible for bonding the undissolved particles to the final structure of the alkali-activated products [83].

Table 3 shows the most relevant mixtures produced by alkaline-activated VA materials [32,38,41,43,44,47,52]. The synthesis parameters include the SiO_2_/Al_2_O_3_ ratio; the type and nature of the activators (i.e., solid or liquid); the concentration and dosage of the activators; and the conditions of the curing regime. The effect of these parameters on the properties of alkaline-activated VA materials, paste, mortar and concretes are described in the following sections.

### 4.1. Type and Dosage of Alkaline Activators

The main alkaline activator is NaOH, which causes a faster reaction with a higher dissolution of aluminosilicate solid precursors and silicate monomers. However, KOH improves the degree of polycondensation and tends to produce stronger matrixes [44,90]. The optimal concentration of these activators is based on their properties and economics. It is possible to say that the molarities of KOH and NaOH used ranges from 5 to 12 M for the activation of VAs. In addition, a high dosage of alkali concentration can cause detrimental effects, such as efflorescence and brittleness, due to the presence of high amounts of free OH^−^ in the final product. Thus, increasing the alkali dosage above 12 M is not recommended.

The silica modulus also influences the mechanical properties of mixtures. Silicates are commercially produced with a SiO_2_/Na_2_O ratio value between 1.5 and 3.2. In general, high-ratio silicates are most suitable for chemical bonding because the siliceous portion of the silicate reacts with the cations. A low-ratio modulus, that is, <2.0, should only be used if the alkaline activation in the mixture is insufficient [91].

According to Bondar et al. [43,44], the activation of VAs with KOH achieved a higher compressive strength than NaOH. However, from an economic perspective, the use of a NaOH activator may be more desirable than a KOH activator, especially under accelerated curing conditions. The pastes produced using VA–type 1 material with high amounts of soluble CaO, and 5 M NaOH and 7.5 M KOH, as the activators had the same compressive strength of 44.0 MPa at 28 days as that produced under autoclave curing. However, VA–type 2 material with high amounts of soluble silicates activated with 7.5 M KOH achieved the best compressive strength of 56.2 MPa at 28 days and curing at 60 °C. Furthermore, the addition of sodium water-glass (i.e., sodium silicate in solution) to a 7.5 M KOH solution increased the effectiveness of the activator in terms of strength. The compressive strengths of geopolymers produced with untreated VA–type 2 material and calcined VA–type 3 material at 90 days, were 49.7 and 39.8 MPa, respectively, both with a SiO_2_/Na_2_O ratio of 3.1. Waterglass with a silica modulus ratio of 3.1 was a suitable activator for a VA with a high CaO content or that had been calcined [44].

Moon et al. [41] confirmed that activators with 80 wt.% NH (10 M NH) and 20 wt.% NS worked adequately and achieved an increase in the compressive strength and a denser microstructure than those of the mixture produced with the NH activator. The analysis of microstructure showed the presence of phillipsite zeolite and C–S–H-like crystal. In this study, the C–S–H product was found in both samples, despite a small amount of Ca in the raw VA (8.8 wt.% CaO; see Table 1). Furthermore, the addition of sodium silicate (NS) accelerated the formation of the C–S–H-like crystal. Thus, it can be suggested that the additional silicates and sodium ions from the sodium silicate play an essential role in the formation of C–S–H. The compressive strengths measured at 28 days were 33 MPa for the VA–NH and 47 MPa for the VA–NH + NS when both samples were submitted to curing conditions of 80 °C and 100% relative humidity.

### 4.2. Conditions of the Curing Regime

High curing temperatures (i.e., 40–90 °C) and extended curing periods increase the rate of dissolution at the early stages, which improve the mechanical and physical properties of alkali-activated VA-based cement [89]. The high curing temperatures induce a higher reactivity and contribute to a rapid setting time for alkali-activated VA-based cement. One study demonstrated that a curing temperature up to 100 °C under atmospheric pressure is the threshold value that could effectively increase the compressive strength of the material [52].

Takeda et al. [47] produced hardened bodies of VA that had an amorphous phase ≤ 30%. The VA was mixed with 10 mL of NaOH solutions at concentrations of 1 M to 16.5 M after curing for 3 days at 50 °C and 80% relative humidity. The maximum compressive strength of the hardened bodies was 80.1 MPa when a 13.5 M NaOH solution was used. Furthermore, a curing time of 3 days was determined to be sufficient, as the compressive strength did not increase after this time. A prolonged period of curing at 50 °C should be avoided to reduce the loss of moisture and the propagation of shrinkage cracks due to water evaporation [58].

Kani and Allahverdi et al. [52] fabricated alkali-activated VA-based paste mixtures with different activators and adjusted molar ratios of SiO_2_/Na_2_O of 0.6 and 0.75, and Na_2_O/Al_2_O_3_ molar ratios of the binder mixture of 0.92, 1.08 and 1.23. These mixtures required a preconditioning process at 25 °C and 95% relative humidity for 7 days before applying the hydrothermal process or autoclave curing to achieve an adequate increase in compressive strength. The alkali-activated mixture samples had a compressive strength of up to 37.5 MPa. Samples that were exposed to the hydrothermal process at 85 °C for 20 h had a compressive strength of 57.5 MPa. The durability of the samples was improved by the formation of more alkali-aluminosilicate gels and the elimination of structural microcracks. The samples that were autoclaved at 210 °C for 30 h had a compressive strength of 108.7 MPa.

The VA material requires a high SSA and a total amount of 36 wt.% of amorphous phases (i.e., SiO_2_ + Al_2_O_3_) to be suitable as alkali-activated pastes. Five alkaline solutions with SiO_2_/Na_2_O molar ratios of 0.7, 0.9, 1.1, 1.3 and 1.4 were prepared and mixed with Na_2_SiO_3_ (modulus of silica, Ms = 1.40) and NaOH (12 M). The samples were then covered with a thin polyethylene film to prevent the evaporation of water and cured at a temperature of 24 ± 3 °C for 24 h before demolding. The VA sample with the greatest SSA of 15.7 m^2^/g and large (Al_2_O_3_ + SiO_2_. content of amorphous phase (37 wt.%), was activated with a SiO_2_/Na_2_O molar ratio of 1.4 and had the highest compressive strength of 50 MPa [32].

Bondar et al. [43] found that an untreated VA sample that had high amounts of soluble silicate (i.e., VA–type 1; see the synthesis parameters in Table 3) was activated with a mixture of 4 mL of 7.5 M KOH solution and 0.5 mL of NS solution with a solid content equal to 2.1%. The VA sample had a compressive strength at 28 days of 81.55 MPa under heat curing at 80 °C. However, after undergoing a calcination treatment at 800 °C and being activated with the same alkaline solution and curing at 20 °C, the same VA material had a compressive strength of 68.5 MPa. Additionally, the researchers verified that untreated VA material with a low LOI, a high CaO content and a soluble SiO_2_/Al_2_O_3_ molar ratio of 4.65 (VA–type 5; see Table 3) was the most suitable pozzolan for activation. Using the same alkaline solution, the untreated VA material produced a paste that had a compressive strength of 53 MPa when cured at 60 °C. It is important to mention that, due to the range of the SiO_2_/Al_2_O_3_ molar ratio, it is improbable that all SiO_2_ and Al_2_O_3_ participate in the synthesis reaction.

### 4.3. Effect of Mineral Additives or Correctors

It is known that VA materials have a lower reactivity than other aluminosilicates materials (i.e., mineral additives), such as MK, fly ash and granulated blast furnace slag (GBFS). The addition of reactive materials that are rich in SiO_2_, Al_2_O_3_ and CaO may help compensate for this deficiency by increasing the reactivity and significantly enhancing the properties of the final products [33,35,45,54]. Moreover, the addition of mineral additives could replace the thermal curing and calcination process used for raw VA materials [27]. Table 4 shows the compressive strengths of different alkali-activated mixtures of VA materials in combination with mineral additives. Although the benefits of adding mineral additives to VA have been verified, mineral additives are not always available. According to Djobo et al. [64], this can contribute to the logistical cost of the product and could compromise the sustainable production in terms of the economics and environmental impact of the use of VA materials on an industrial scale. Therefore, alternative and locally available mineral additives must be considered.

Bondar et al. [45] studied the effect that active mineral additives, such as kaolinite, burnt lime and calcined VA, had on the initial composition of raw VA before alkaline activation. The mineral additives contain readily available Al, Si and Ca that can compensate for the lack of these species in the initial raw VA material and enhance the composition of the material. The produced mixtures contained up to 40% kaolinite, 100% calcined VA and 7% burnt lime; a 7.5 M KOH solution was used as the activator in all the mixtures (see the synthesis parameters in Table 4). The compressive strengths of the alkali-activated VA-based pastes without mineral additives were 44 and 19.48 MPa when autoclaved at 2 MPa and 150 °C for 3 h and sealed for curing at 25 °C, respectively. At 28 days, the strength of the binder pastes from the mixture made with 20 wt.% kaolinite increased up to 13% and 4.5% for autoclaved and sealed curing at 25 °C, respectively, compared with the VA without additives. The addition of 16.7% calcined VA increased the compressive strength of the mixture up to 11% and 10.5% for autoclaved and sealed curing at 25 °C, respectively, compared with the VA without additives. The AAM materials cured by autoclave had a higher strength than samples sealed cured when kaolinite and calcined VA were added. The average molar oxide ratios of SiO_2_/Al_2_O_3_ for the final products were between 3.3 and 6.5, which are within the range that favours alkaline activation.

Tchakouté et al. [34,35] analysed the effect of the addition of Al_2_O_3_ on the characteristics of alkali-activated pastes made from VA. The results showed that an addition of up to 40% Al_2_O_3_ increased the compressive strength by 32.4% (47.8 MPa) with respect to that of paste produced with 100% VA (36.1 MPa). However, the researchers showed that excess Al_2_O_3_ (i.e., >40%) exhibited a non-homogeneous microstructure with cracks. Additionally, Tchakouté et al. [34,35] used the alkali-fusion process to enhance the reactivity of VA; the process involved thoroughly mixing the VA material with NaOH pellets at a low alkali/VA mass ratio of 0.7, followed by the addition of highly reactive MK to consume the excess alkali needed for the fusion. The amount of the reactive phase in the VA after the alkali-fusion process was higher (76%) than that in raw VA (26%). The mortars were prepared by alkali activation of mixtures of powders of fused VA (f-VA), various amounts of MK and river sand at f-VA/MK mass ratios of 70/30, 60/40, 50/50 and 40/60 using a sodium silicate solution as the activator. The produced mixture had a compressive strength of 68.8 MPa at 28 days of curing for the f-VA/MK ratio of 40/60.

Djobo et al. [84] obtained similar results. The researchers used various amounts of MK (i.e., 5 wt.%, 10 wt.%, 15 wt.%, 20 wt.% and 25 wt.%) to compensate for the deficiency of Al_2_O_3_ and to increase the amount of the amorphous phase in the VA. The addition of 25% MK in the VA increased the amount of amorphous Al_2_O_3_ and SiO_2_ in the system. A high-alkaline solution with a SiO_2_/Na_2_O ratio of 1.4 favoured the dissolution of these species and promoted the formation of an alkali-activated paste with a compressive strength of 69 MPa and a decreased setting time.

Robayo-Salazar et al. [27] verified that the low reactivity of VA (amorphous phase content of 25.5%) required the application of a thermal curing at 70 °C to harden and develop the strength to early curing ages. However, the incorporation as a replacement for GBFS in the mixture in amounts up to 30 wt.% to VA made thermal curing unnecessary during the binder synthesis process. A water solution of the alkaline activators of NaOH and Na_2_SiO_3_ with SiO_2_/Al_2_O_3_ ratios of 6.5–7.0 was used in the synthesis process of the binder (see synthesis parameters in Table 4). The binder had a compressive strength of up to 125 MPa at 28 days of curing at a room temperature of 25 °C. In contrast, the use of NaOH (5% Na_2_O) as an activator without Na_2_SiO_3_ required a curing process of 2 days at 70 °C to achieve a compressive strength of 30 MPa at 28 days. The Scanning Electron Microscopy-Energy Dispersive X-ray Spectroscopy (SEM/EDS) analysis verified the production of C-S-H gels with a high calcium composition and N-A-S-H gels with a low calcium composition. Over time, the interaction of the N-A-S-H and C-S-H gels led to the formation of gels with an intermediate composition type (C, N)-A-S-H and C-A-S-H that had higher contents of calcium and aluminium [42]. The formation of these phase gels caused a decrease in pore sizes as well as in the total pore volume, from 12.6% in a mixture of 100% VA to 6.2% in a mixture of 70% VA–30% GBFS. These phase gels had a better mechanical performance compared with the performance of 100% VA paste.

Vafei and Allahverdi [54] studied the applicability of calcium aluminate cement (CAC) as a mineral additive. They verified that mixtures produced with CAC in 24 wt.% of total binder with a modulus of silica of 1.5 and 10% Na_2_O had up to 65 MPa of compressive strength under hydrothermal curing conditions. Under the same conditions, a 100% VA mixture had a compressive strength < 20 MPa. The workability and setting time of the produced mixture was reduced by increasing the CAC content.

## 5. Fresh and Hardened Properties of Alkali-Activated Volcanic Ash-Based Pastes and Mortars

The use of alkali-activated VA-based materials in mortar production improves the fresh state properties of workability, mobility, placeability and water retention compared with OPC mortars. In addition, using alkali-activated VAs reduces the permeability of mortars and improves their durability properties, as well as their resistance to sulphate and chloride attacks, and even surpasses the properties of OPC mortars [22,36,46,55]. Table 5 gives a summary of the representative studies on alkali-activated VA-based mortars produced with an aggregate/binder ratio of 2 [22,46,55].

### 5.1. Fresh Properties

#### Setting Time and Heat Hydration

The setting time of alkali-activated pastes depends on the particle size distribution and the chemical composition of the raw VA materials. The low SSA and free CaO content in the source material can lead to higher setting times for alkali-activated blends [19]. The setting time of alkaline pastes produced using VA material with a high amount of CaO (5.11 wt.%) and a high SSA of 15.7 m^2^/g decreased from 490 min to 180 min when the silica modulus of the alkaline solution was modified from 0.7 to 1.4 [32]. Djobo et al. [84] determined that when the VA material was used with MK and an alkaline solution with a high SiO_2_/Na_2_O molar ratio, there was a reduction in the setting time of the produced paste. The paste mixture produced with an alkali solution with a silica modulus SiO_2_/Na_2_O of 1.4 had a setting time of 180 min.

Geopolymer pastes produced with VA materials activated by MA showed a reduction in the initial and final setting times. Djobo et al. [38] found that a milling process time of 60 min increased the fineness of the VA particles and caused a reduction in the setting time of up to 95%.

Mineral additives also influence the setting time of AAMs. Lemougna et al. [92] used 10% GBFS to replace VA materials and found that it reduced the setting time from 7 days to 6.7 h when the test was carried out at 25 °C. Robayo-Salazar et al. [27] also reported that the setting time of an alkaline-activated paste was reduced when NH and NS was used and GBFS replaced some of the VA materials during production. Although the pastes that contained 100% VA needed > 48 h for setting, the paste that contained 30% GBFS and 70% VA needed an initial and final setting time of 22 and 30 min, respectively. In addition, the mixture presented a total heat of reaction of up to 48 h (44.48 J/g), a value that is 76% less than the paste produced using 100% OPC (190 J/g).

**Figure 4:**
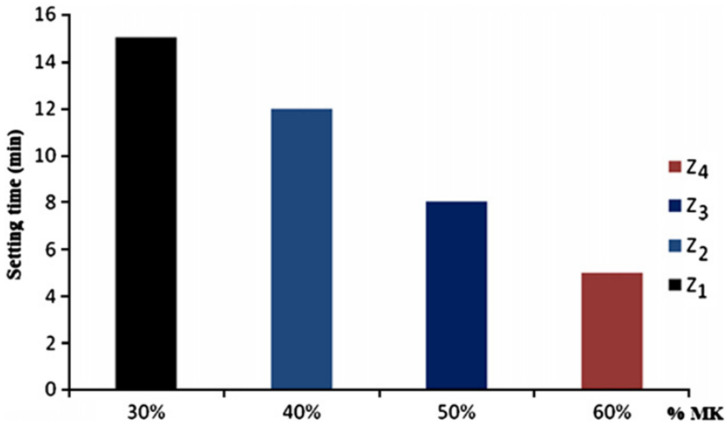
Setting time of fused alkali-activated mortars. The mortars obtained with 30%, 40%, 50% and 60% MK were labelled as Z1, Z2, Z3 and Z4, respectively [34].

Tchakouté et al. [34] studied the mortar blends produced using an alkaline-activated (sodium silicate solution) composed of VA materials to MK ratios of 70/30, 60/40, 50/50 and 40/60. It was determined that the setting time decreased when up to 60% of MK was used in the mixture production (see Figure 4). The high amount of MK increased the presence of Al and Si species, which increased the formation of a polymeric binder, compared with a mixture that contained a low amount of MK.

### 5.2. Density, Apparent Porosity and Water Absorption

Djobo et al. [22] reported that mixtures cured at 27 and 80 °C achieved similar density values around 2166 kg/cm^3^; all synthesis parameters and cured conditions are described in Table 5. However, water absorption and apparent porosity values were lower for specimens cured at 80 °C than for specimens cured at 27 °C. Consequently, the researchers concluded that a high degree of geopolymerization was achieved when the samples were cured at a high temperature. The maximum values of water absorption were 7.03% and 5.91% after 28 days curing at 27 and 80 °C, respectively.

Lemougna et al. [36] produced mortar samples with a composition Na_2_O/SiO_2_ molar ratio of 0.3. Although the VA-based mortars made using 10 wt.% sand had the lowest bulk density of 1910 kg/m^3^ and the highest water absorption of 14.61%, the mortars produced with 25 and 40 wt.% sand had a higher density and a lower absorption capacity. The addition of a sand filler can reduce manufacturing costs provided that it is not added in larger proportions than those required to maintain the appropriate physical properties for the intended application.

According to Ghafoori et al. [46], a mixture produced with a high hydroxide concentration with a greater alkaline activation, as well as a reduction in the solution-to-binder (S/B) ratios, produced a more compact matrix with a decrease in the absorption capacity of the mixture. The researchers found that the absorption reduced from 13.2% to 2.4% when the molarity of the solution increased from 2.5 to 12.5 M. In addition, the porosity reduced by 37% when the molarity of the solution increased from 2.5 to 12.5 M.

### 5.3. Compressive Strength of Mortars

Ghafoori et al. [46] produced mortar samples with an S/B ratio of 0.58, a 12.5 M NaOH solution and an aggregates/binder ratio of 2 (see Table 5). After exposed (dry), wet and sealed curing conditions were applied at 80 °C, the mortars achieved a compressive strength of 31.8, 26.6 and 37.7 MPa at 7 days, respectively. The mortars that were exposed to the sealed curing condition achieved the highest compressive strength, while mortars exposed to the wet curing condition had the lowest strength. Similarly, Kantarci et al. [55] showed that a maximum compressive strength of 37 MPa at 90 days occurred when the mortar samples were exposed to sealed curing for 72 h at 120 °C. After the curing process was completed, the samples were kept under laboratory conditions until test day. In the study, the mortar samples were produced using alkaline activation of VA with 16 M NaOH solution, an S/B ratio of 0.45 and an aggregates/binder ratio of 2. However, the researchers found that the curing process at high temperatures may cause the gel structure of the geopolymer to break down due to excessive shrinkage.

Djobo et al. [22] also found that mortar samples cured at 80 °C had a higher compressive strength of 38 MPa at 90 days compared with samples cured at 27 °C. The high-temperature curing process achieved highly cross-linked AAM materials and an adequate interfacial transition zone between the paste and the aggregates, which increased the compressive strength of the geopolymer mortar [93]. Because the bonding strength of a geopolymer gel is related to the extent of geopolymerization, it was greater in specimens that were cured at 80 °C than in specimens cured at 27 °C.

According to Tchakouté et al. [34], the use of MK as a mineral additive improved the hardened properties of alkali-activated VA-based mortars. The mortars were prepared by activating blends of fused VA, MK and river sand in a ratio of 40/60 by sodium silicate solution (see Table 4). The aggregate-to-binder ratio was set at 2 by weight. MK has a small particle size of 9.95 µm and a high SSA of 20.5 m^2^/g, which improved the polycondensation phenomena and the formation of a polymeric binder. Thus, the compressive strength of these binders increased to 68.8 MPa (see Table 4).

**Figure 5:**
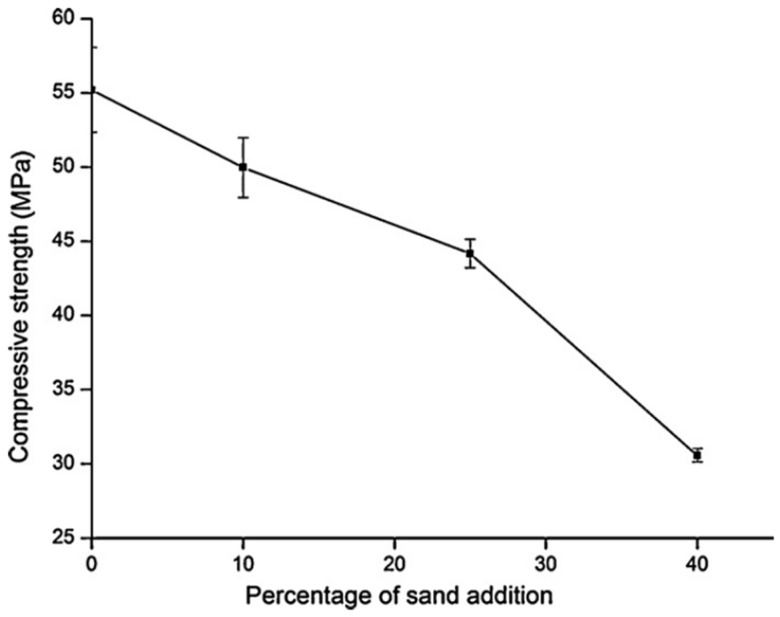
Influence of the addition of mortar on the compressive strength of a mortar with a molar composition of Na_2_O/SiO_2_ = 0.30 [36].

The research work carried out by Lemougna et al. [36] was based on the production of mortar for structural and refractory applications. The researchers demonstrated that the addition of sand decreased the porosity and increased the bulk density of the alkali-activated mortar. However, the compressive strength decreased from 55 to 30 MPa when the proportion of sand increased from 10% to 40% with respect to the VA weight (see Figure 5). The reported compressive strength of 30 MPa with 40% of sand met the requirements of ASTM C216 for severe weathering for building materials. In addition, the increase in sand employment from 10% to 40% reduced the standard deviation of the compressive strength from 2.5 MPa to 0.5 MPa, respectively (see Figure 5), and the use of sand also reduces the costs involved in manufacturing.

## 6. Engineering Properties of Alkali-Activated Volcanic Ash-Based Concretes

Several researchers highlighted the potential of using raw VA material to obtain alkali-activated concretes [5,57,58,94]. Table 6 shows the concrete mixture proportions for alkali-activated concretes. The mixture proportions and curing conditions of the concretes are given in Table 7. 

Haddad and Alshbuol [58] produced alkali-activated concretes using 100% VA material as a binder. The mixture contained coarse limestone, fine aggregates with silica sand, a water-to-binder (w/b) ratio of 0.40 and a superplasticizer (see Table 6). Different sodium silicate to sodium hydroxide (NS/NH) by weight ratio were used as alkali activators in combination with normal- and high-temperature curing processes. The NS was composed of 29 wt.% SiO_2_, 11 wt.% Na_2_O and 60 wt.% H_2_O; 14 M sodium hydroxide (NH) was also used. The concrete produced with the 2.5 NS/NH activator had adequate fresh properties. In addition, the highest compressive strength of 31.9 MPa was achieved under a dry curing period of 24 h at 80 °C. The strength test values were very close, as indicated by the standard deviation of about 4%. Curing temperatures >80 °C were detrimental to the compressive strength of the alkali-activated VA concretes. The researchers used SEM images to verify that the aluminosilicate gel that was formed was dense with continuous, but limited, microcracks. These results confirmed the behavioural trend that was observed for compressive strength.

Bondar et al. [94] found that alkali-activated VA-based concretes had lower compressive and tensile strengths and modulus elasticity compared with OPC mixtures at early stages. However, the activated VA material with a w/b ratio of 0.45 and calcined VA under sealed curing at 40 and 20 °C achieved the same and even higher values of mechanical properties than OPC mixtures after long-term aging (after 365 days): up to 15% in compressive strength and up to 20% in modulus of elasticity, respectively. The compressive strength and modulus elasticity achieved 43.5 MPa and 33.6 GPa values, respectively. However, the activated VA mixture with a w/b ratio of 0.42 under sealed curing at 60 °C showed a much lower value of static modulus of elasticity and strength than the OPC mixtures. While the VA mixtures achieved a value of 10.7 GPa and 35 MPa in modulus elasticity and compressive strength, the OPC obtained 29 GPa and 38 MPa, respectively. The high curing temperature of 60 °C caused water evaporation and incomplete alkaline activation, reducing the compressive strength and static modulus values.

Ibrahim et al. [57] reported that alkali-activated VA-based concretes that were produced using 300–450 kg/m^3^ of VA material and a NS/NH sodium silicate to sodium hydroxide by weight ratio of 2.0–2.75 had a reduced compressive strength after sealed concrete samples had a prolonged exposure to 60 °C. However, the sealed concrete samples achieved a significant gain in strength after 3 days or 7 days of curing at 60 °C. The concrete produced with 400 kg/m^3^ of binder and a NS/NH ratio of 2.5 under a sealed curing process at 60 °C for 7 days achieved the highest compressive strength of up to 37.5 MPa, which was suitable for construction applications.

Robayo-Salazar et al. [5] also verified the use of alkali-activated VA-based concretes. They mixed 70% VA and 30% GBFS as the binder during concrete production. Compared with OPC concrete, the mixed concrete achieved an equal or higher compressive strength of 33 MPa. In addition, the alkali-activated VA–GBFS concretes had a 44.7% lower carbon footprint: the alkali-activated concrete = 210.90 kg CO_2_ eq/m^3^ versus OPC concrete = 381.17 kg CO_2_ eq/m^3^. Thus, alkali-activated VA-based concretes represents an alternative option to traditional OPC concrete.

## 7. Durability Properties of Alkali-Activated Volcanic Ash-Based Materials

Few studies have focussed on assessing the durability of alkali-activated VA-based cements/concretes [22,46,94,95,96,97]. Alkali-activated blended materials have demonstrated adequate durability properties in terms of oxygen and chloride permeability, sulphate and sulfuric acid corrosion, drying shrinkage/crack and efflorescence.

### 7.1. Permeability

Bondar et al. [96] investigated the oxygen and chloride permeability of two Iranian VAs and accounted for different synthesis conditions. The researchers found that, under sealed conditions and high curing temperatures of 40 and 60 °C, the oxygen permeability decreased for mixtures with a low w/b ratio; the mixtures produced with a w/b ratio of 0.42 had the lowest permeability compared with mixtures produced with a w/b ratio of 0.45 or 0.55. Additionally, the alkali-activated concretes exhibited up to 35% lower oxygen permeability than that of OPC concrete at 90 days. The chloride penetrability was also found to be lower after the sealed and heated curing process at 40–60 °C.

According to Ghafoori et al. [46], the chloride penetration depth of alkali-activated VA-based mortars was reduced from 45.62 to 4.49 mm when the concentration of NaOH solution increased from 2.5 to 12.5 M. In addition, the chloride penetration depth was reduced up to 14.1% and 11.4% when the sodium hydroxide S/B ratio was reduced from 0.58 to 0.54 and 0.54 to 0.50, respectively.

### 7.2. Sulphate and Acid Resistance

The w/b ratio is a significant factor that affects the sulphate resistance of alkali-activated VA-based concrete. A small w/b ratio results in a lower weight loss in concretes after a sulphate resistance test. For example, Bondar et al. [95] reported on the compressive strength of alkali-activated natural pozzolan (AANP) concrete samples after sulphate exposure. The samples were immersed for 2 years in a solution of 2.5 wt.% Na_2_SO_4_ and 2.5 wt.% MgSO_4_. The compressive strength of the immersed samples was 8–19.5% less than the strength of the identical samples cured outside of the solution. However, the maximum percentage of expansion by the AANP mixture was 0.086% at 6 months, and the trend was for this to decrease with time. Therefore, the maximum percentage expansion measured for the AANP concrete was <0.1%, which is acceptable according to the ASTM C1012 Standard for OPC concretes with moderate sulphate exposure conditions at 6 months.

**Figure 6:**
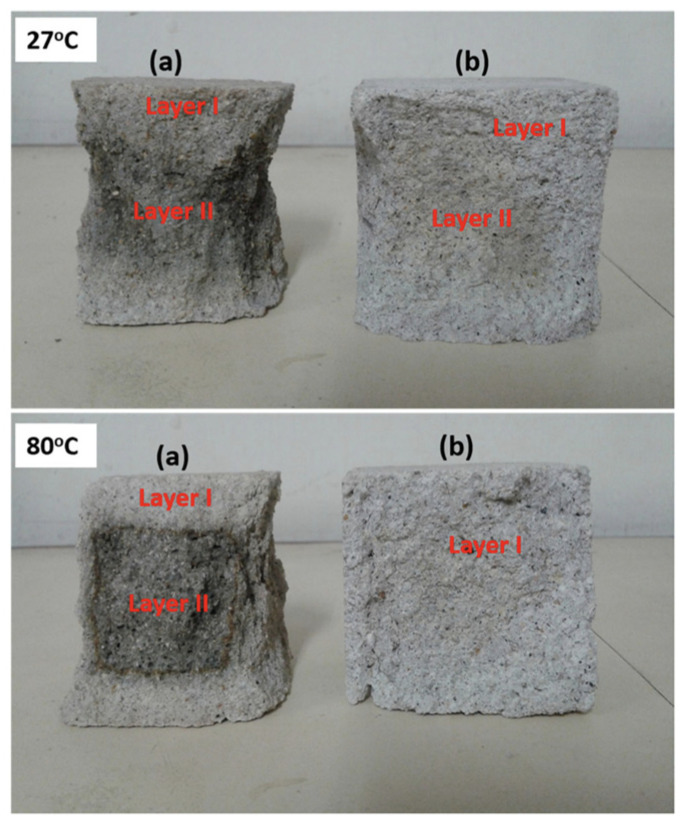
Visual aspect of the core of the alkali-activated VA-based mortars after (**a**) 90 days and (**b**) 180 days of exposure to 5% H_2_SO_4_ [22].

Djobo et al. [22] studied the acid resistance of alkali-activated VA-based mortars using 5% H_2_SO_4_. The synthesis parameters (Table 5) showed that the samples sealed curing conditions at 27 °C developed better acid resistance than the samples heat-cured at 80 °C. The presence of a Na-rich gel in a sample cured at 27 °C lowered the pH of the system through an acid–base reaction and slowed the gypsum formation process. In addition, samples cured at 27 and 80 °C showed a decrease in compressive strength of 24% and 60%, respectively, after 180 days of exposure to the acid solution. The researchers determined that samples cured at 27 °C showed very tiny pores or had lower connectivity between pores than samples cured at 80 °C. Figure 6 shows the visual aspect of the alkali-activated VA-based mortar samples cured at 27 and 80 °C after exposure to the acid solution. Both cured samples showed a low weight-loss value of 3–3.5%, which could be attributed to the stability of the aluminosilicate framework of the alkali-activated VA-based mortar.

### 7.3. Drying Shrinkage/Crack

According to Bondar et al. [94], the curing regime and time applied in the synthesis of alkali-activated VA-based concrete affect the amount of drying shrinkage. Samples under sealed curing conditions of 60 °C for 3 days, as well as samples with a high w/b ratio of 0.55 under sealed curing conditions for 7 days, had the lowest drying shrinkage values. The maximum final drying shrinkage value at 180 days for a sample mixture with a w/b ratio of 0.55 was 514 × 10^−6^, which was 43% lower than that of the sample mixed with a w/b ratio of 0.45 (1185 × 10^−6^). The researchers determined that the cross-linking in the samples with the lowest w/b ratio was not efficient, as the loss of moisture from fresh alkali-activated VA concrete resulted in a reduction in volume, so it was possible that the cross-linking in the sample was not complete. Additionally, the reduction in drying shrinkage at high temperatures is related to the removal of water and further cross-linking of hydration products. Fog curing at 40 °C showed a higher amount of drying shrinkage at 3 days and 7 days that was related to the retention of water by the alkali-activated binder matrix, which produced a more porous microstructure in this type of concrete [98].

### 7.4. Efflorescence

Efflorescence occurs when free alkalis of the pore solution react with humid air containing CO_2_ and form white deposits on the surface of the concrete structure that are characteristic of carbonate compounds, such as Na_2_CO_3_ or K_2_CO_3_. Allahverdi et al. [97] showed that the severity of efflorescence can be controlled by optimizing the composition of the alkaline activator and the w/b ratio. They observed no or a slight efflorescence appearance in samples that had an alkaline activator consisting of 4 wt.% Na_2_O, a modulus of silica ranging from 0.52 to 0.68 and a w/b ratio varying from 0.36 to 0.44. However, these samples did not exhibit an acceptable 28-day compressive strength, that was comparable to samples that contained 10 wt.% Na_2_O, which exhibited severe efflorescence.

Further studies have demonstrated that efflorescence in alkali-activated VA-based binders can be reduced by adding an appropriate amount of active aluminum into the system using minerals such as MK, GBFS and CAC [35,53,84]. Efflorescence can also be reduced by enhancing the release of aluminum from a less active precursor by adjusting the curing conditions. The use of hydrothermal curing at a temperature of 65 °C or higher can also mitigate the extent of efflorescence and causes a slight increase in the compressive strength of the samples [52,97].

## 8. Conclusions

The following conclusions have been obtained from the present review study about the VA properties and activity:

- VA is an unexploited suitable and sustainable raw material that is deposited around the world; VA has easy accessibility, low cost and little environmental impact;
- For alkali-activated binder production, VA material should have a composition of SiO_2_ + Al_2_O_3_ ≥ 70%. Additionally, the optimum SiO_2_/Al_2_O_3_ molar ratio should be 3.3 to 4.5 and the amorphous content should be a minimum of 36% to be suitable for alkali-activated binder production;
- MA and calcination increase the VA reactivity. MA increases the SSA of VA and reduces the crystallinity. However, an extended grinding process could cause a reduction in VA reactivity. A calcination process >700 °C can cause the crystalline phases to become an amorphous and more reactive material.

The following conclusions can be made about the effects of alkaline activation and the curing condition:

- Due to the weak reactivity of VA in an alkaline environment, a high concentration of 10–14 M of an alkali activator is required. Strong bases, such as NaOH (NH), KOH (KH) and Na_2_SiO_3_ (NS), have been used. NH causes a faster reaction and a higher dissolution of VA. KH improves the degree of polycondensation and produces stronger matrixes. Both NH and KH, in combination with NS, cause a faster strength development, and thus a higher ultimate strength of AAM materials. However, increasing the alkali dosage can produce detrimental effects, such as efflorescence, due to high amounts of free alkali in the final product;
- High curing temperatures of 40–90 °C under atmospheric pressure in sealed curing conditions and extended curing periods lead to an increase in the dissolution rate (i.e., synthesis of AAM) at early stages. This can improve the mechanical and physical properties of AAM. A long-term heat-curing period of 7 days could improve the microstructural refinement in the matrix and restrict water loss during the drying period, which could help mitigate binder shrinkage. However, prolonged curing at high temperatures could also cause shrinkage cracks.

The following conclusions are presented about the effect of mineral additives on reactivity:

- Adding mineral additives, such as MK, GBFS and lime, can compensate for deficiencies of the main oxides, such as SiO_2_, Al_2_O_3_ and CaO, in VA materials. These mineral additives increase the level of oxides in the final product before activation. Adding small fractions (5–30%) of minerals, especially MK or GBFS, reduce the setting times and increase the heat of reaction and the strength of these binders at an early stage. Furthermore, these minerals could replace hydrothermal curing during binder synthesis and calcination processes >700 °C for VA with low reactivity;

The following conclusions can be made about fresh and hardened properties of alkali-activated VA-based paste and mortar:

- Adequate fresh properties of setting time and fluidity can be achieved using proper combinations of mixture proportions. In this case, the concentration of the activators, such as NaOH, and the S/B ratio are the most important factors. The workability of the paste decreases when a concentration of NaOH > 12 M and an S/B ratio < 0.4 are used;
- The mortar mixtures produced with a high NaOH concentration between 10–12 M and with a low S/B ratio of 0.4 have an increased mechanical performance in terms of strength;
- Increasing the S/B ratio decreases the compressive strength because of the higher liquid content. Increasing the S/B ratio beyond a certain point, particularly for alkali-activated mortars with S/B ratios of 0.45–0.65, will not increase the strength, although it will enhance the workability. Mortar mixes prepared with a low S/B ratio are less workable;
- The alkali-activated VA-based mortars exhibit good performance under wetting and drying cycles. In addition, the presence of Na-rich gel improves the acid resistance. However, the structure and permeability of the pores are key factors in controlling their durability properties.

The following conclusions are presented about alkali-activated VA-based concrete:

- The few studies that have been published on alkali-activated VA-based concrete show that the concrete has good workability and compressive strength development when the materials are produced at acceptably low w/b ratios of 0.42–0.45;
- While the alkali-activated VA-based concretes mostly have low strength and modulus of elasticity than OPC mixtures at early stages, they reach the same or higher strength and modulus of elasticity after long-term curing;
- Information about the durability properties of alkali-activated VA-based concrete/materials as an environmentally friendly construction material is limited.

From various studies, it is clear that the production of VA-based alkali-activated materials as a building material is promising but still has challenges to be overcome prior to scale-up. Firstly, the synthetic approach in terms of origin, availability, and logistics of precursors and alkaline activators with high purity and a wide Si/Al ratio range for possible astringent applications needs to be optimized. Secondly, there are no studies with alternative activators with low environmental impact and cost compared to NaOH/KOH or Na_2_SiO_3_ activators currently used. Studies of hybrid materials, where high VA contents and low percentages of Portland cement are used, should be investigated. Thirdly, because of the chemical differences between OPC and VA-based alkali-activated materials, rigorous research and a deep understanding of surface chemical phenomena in these systems are required. Fourthly, the existing structural design codes for Portland concrete are based on a set of implicit assumptions related to its microstructure and macro-behaviour under different environmental conditions. Although some researchers have reported the engineering properties of VA-based alkali-activated concretes, these assumptions may not be valid in the case of these materials. Consequently, there is a generalizable lack of knowledge about the behaviour of long-term VA-based alkali-activated materials. Research into these topics must be considered in the near future.

## Figures and Tables

**Table 1:** Chemical composition of VAs from different investigations.

Origin and Type Reference	SiO_2_ (%)	Al_2_O_3_ (%)	Fe_2_O_3_ (%)	CaO (%)	MgO (%)	Na_2_O (%)	K_2_O (%)	LOI (%)
Cameroon (Ndjock et al. [31])	40.1	16.3	13.5	6.30	5.3	0.8	0.5	11.1
	45.8	13.8	13.5	11.8	8.4	3.2	1.5	0.1
	45.3	12.4	12.9	13.1	9.9	2.4	1.3	0.2
	46.1	14.0	13.1	10.7	7.0	2.7	1.4	1.5
	45.5	12.6	13.3	13.4	9.5	2.9	0.9	0.9
Cameroon Basaltic (Tchakouté et al. [32,33,34,35])	41.36	15.41	12.88	7.88	6.45	2.22	0.90	9.31
	44.04	15.26	12.77	9.29	7.0	5.64	1.35	1.1
Cameroon Basaltic (Lemougna et al. [36,37])	44.19	14.06	13.22	10.38	9.73	3.69	1.53	−0.62
	43.0	15.0	12.0	11.0	6.80	4.6	1.7	–
Cameroon Basaltic (Djobo et al. [22,38,39])	46.28	15.41	13.22	9.07	6.74	3.88	1.42	−0.40
	47.74	15.36	12.88	8.25	6.95	3.62	1.11	0.66
Papua New Guinea Andesite (Hossain [40])	59.32	17.54	7.06	6.10	2.55	3.80	2.03	1.03
Saudi Arabia Basaltic (Moon et al. [41])	46.48	14.74	12.16	8.78	8.73	3.39	1.27	1.32
Colombia Andesite (Robayo el al. [5,27,42])	61.99	15.52	7.33	5.19	2.49	4.07	1.59	0.48
	61.17	16.57	5.81	2.86	3.73	0.66	0.68	7.63
Iran Dacite and Andesite (the last sample) (Bondar et al. [43,44,45])	70.13	11.11	1.27	2.52	0.92	1.01	2.25	10.28
	64.67	11.85	3.03	6.79	1.11	2.30	4.26	5.15
	68.51	11.84	3.73	2.90	1.43	1.62	3.19	6.14
	68.31	12.59	2.70	3.88	1.37	2.40	3.26	4.41
	61.67	15.90	4.32	7.99	2.04	3.21	2.12	1.85
USA Dacite (Ghafoori et al. [46])	68.80	8.50	3.80	3.20	–	5.20	–	3.7
Japan Basaltic Andesite (Takeda et al. [47])	54.93	16.44	10.89	8.79	3.30	2.84	1.72	–
Algeria Amphibole-biotite Andesite and Rhyodacite (Labbaci et al. [48])	51.88	16.31	8.81	6.12	3.98	3.93	3.09	2.38
	67.21	13.53	5.96	0.57	1.05	3.48	5.44	1.4
Algeria Andecite (Hamidi et al. [49])	57.3	15.0	6.0	9.0	2.0	1.8	1.2	3.3
Saudi Arabia (Kunal et al. [50])	47.0	14.8	12.5	9.29	7.95	3.54	1.4	–
Turkey Pumice (Kılıc and Sertabipoglu [51])	72.58	14.49	2.14	1.08	0.11	4.03	4.92	–
Iran Pumice (Kani and Allahverdi [52,53], Vafaei and Allahverdi [54])	61.57	18.00	4.93	6.69	2.63	1.65	1.95	2.15
Turkey Tuff (Kantarci et al. [55])	77.22	18.89	1.77	0.27	–	–	0.91	–
Saudi Arabia Al-Fadala et al. [56]	44.54	13.45	12.18	9.28	8.11	3.79	1.35	1.32
Turkey (Ibrahim et al. [57])	40.48	12.90	17.62	11.83	8.33	3.60	1.67	1.6
Jordania (Haddad and Alshbuol [58])	40.17	13.86	15.16	9.7	9.57	3.66	1.53	4.75
Greece Tuff (Moropoulou et al. [59])	69.66	12.21	2.34	2.01	0.70	0.62	3.28	7.35
General Range	40–70	10–20	1–15	2–10	1–10	1–5	1–5	<15

**Table 2:** Physical properties of VA obtained by different researchers.

Reference	Fineness/Specific Surface Area (m^2^/kg) (2/kg)	Specific Gravity	Bulk Density (kg/m^3^)
Djobo et al. [22]	–	2.62	–
Djon Li Ndjock et al. [31]	731.9–261.4	–	–
Tchakouté et al. [32]	2300 and 15，700	–	–
Bondar et al. [43]	1062.1–383.6	2.28–2.08	–
Ghafoori et al. [46]	608.8	2.29	–
Kılıc and Sertabipoglu [51]	1640	2.42	895
Vafaei and Allahverdi [54]	380	2.22	–
Al-Fadala et al. [56]	296 and 396	–	2750–2780
Hossain [61]	242	–	2450 *
Olawuyi and Olusola [62]	–	3.04	1649 **

* Oven dry basis; ** Compacted bulk density.

**Table 3:** Summary of the most relevant synthesis parameters on alkali-activated volcanic ash (VA)-based pastes.

Raw Material		Types of Activators/Alkaline Solution				Curing Conditions	Compressive Strength	Ref.
VA ^1^		(NS ^2^, NH ^3^, KH ^4^)	SiO_2_/Na_2_O	Na_2_O/SiO_2_	L/S ^5^			
VA (ZD), <80 µm, ≥2.3 m^2^/g, SiO_2_/Al2O_3_ 4.90		NS + NH (12 M)	0.7, 0.9, 1.1, 1.3 and 1.4		0.37	Ambient temp. 24 ± 3 °C for 28 days.	19 MPa at 28 days	Tchakouté et al. [32]
VA (ZG), <80 µm, ≥15.7 m^2^/g, SiO_2_/Al_2_O_3_ 4.55					0.49		50 MPa at 28 days	
Vas < 75 µm:	Type 1 (High soluble CaO)	KH (2.5, 5, 7.5, 10 M) NH (2.5, 5, 7.5, 10 M) NS	2.1, 2.4, and 3.1		3.33	Sealed and cured at 40 °C and 60 °C and Autoclave at 2 MPa and 150 °C for 3 h	44 MPa at 28 days	Bondar et al. [44]
	Type 2 (High soluble silicates)					Sealed and cured at 40 °C and 60 °C	56.2 MPa at 60 °C at 28 days	
							49.7 MPa at 90 days (Ms, 3.1)	
	Type 3 (Type 2 but calcined at 800 °C)					Sealed and cured at 40 °C and 60 °C	39.8 MPa at 90 days (Ms, 3.1)	
VA ≤ 200 µm		NH (1–16.5 M)			0.3	50 °C and 80% RH for 3 days	80.1 MPa at 3 days	Takeda et al. [47]
Volcanic pumice ground ≥305 m^2^/kg, ≤22.63 µm		NH + NS	0.6 and 0.75	Na_2_O/Al_2_O_3_0.92, 1.08, 1.23		Precuring: 95% RH at 25 °C Hydrothermal treatment in steam saturated atmosphere at 45, 65, 85 °C for 5, 10, 15 and 20 h after 1 and 7 days of precuring	37.5 MPa at 28 days after 1-day of precuring	Kani & Allahverdi [52]
							57.5 MPa at 28 days after 7-day of precuring	
		NH +NS	0.6	Na_2_O/Al_2_O_3_1.08		Autoclave curing at 125, 150, 180 and 210 °C for time periods of 20, 30, 40 and 50 h	108.75 MPa	
VA < 75 µm		NH (10 M)	3.22		0.45	Oven at 80 °C and 100% RH cured for 1, 3, 7 and 28 days	33 MPa at 28 days	Moon et al. [41]
		80% NH (10 M) + 20% NS					47 MPa at 28 days	
VA < 75 µm VAs untreated and calcined at 700, 800, 900 °C	Type 1	KH (5–7.5 M) + NS	2.1			Curing temperature of 20, 40, 60 and 80 °C for 27 days.	68.5 MPa calcined at 800 °C and cured at 20 °C 81.55 MPa untreated and cured at 80 °C	Bondar et al. [43]
	Type 2						32.9 MPa calcined at 900 °C and cured at 80 °C 8.0 MPa untreated and cured at 80 °C	
	Type 3						13 MPa calcined at 800 °C and cured at 80 °C 29 MPa untreated and cured at 60 °C	
	Type 4						42.4 MPa calcined at 800 °C and cured at 80 °C 22.3 MPa untreated and cured at 60 °C	
	Type 5						65 MPa calcined at 700 °C cured at 80 °C 53 MPa cured at 60 °C	
VA < 200 µm and mechanical activation, milling time	60 min	NS + NH (12 M) NS/NH (mass ratio) = 2.4	1.45		0.40	Cured at 27, 45 and 60 °C for 24 h then demolded and cured at room temperature	27 °C: 32.1 MPa; 45 °C: 34.5 MPa; 60 °C: 29.4	Djobo et al. [38]
	90 min						27 °C: 37 MPa; 45 °C: 52.5 MPa; 60 °C: 48.3	
	120 min						27 °C: 45.8 MPa; 45 °C: 53.6 MPa; 60 °C: 46.8	

^1^ Volcanic ash; ^2^ Na_2_SiO_3_; ^3^ NaOH; ^4^ KOH; ^5^ Liquid-to-solid ratio.

**Table 4:** Summary of the effect of the addition of mineral additives and correctors to activate VA.

Raw Material (VA ^1^)	Product	Types of Activators/ Alkaline Solution (KH ^2^, NS ^3^, NH ^4^, Ms ^5^)	Additive	Curing Conditions	Compressive Strength (28 Days)	Optimal Conditions	Ref.
Iran VA	Paste	KH (7.5 M) + NS SiO_2_/Al_2_O_3_ = 3.3 & 6.5 for final products	Kaolinite	Autoclave	45.61 MPa	20% kaolinite	Bondar et al. [45]
				Sealed cured at 25 °C	19.26 MPa		
			Calcined VA (at 800 °C for 12 h)	Autoclave	45.56 MPa	16.7% calcined VA	
				Sealed cured at 25 °C	25.28 MPa		
			Burnt Lime	Autoclave	27.8 MPa	3.4% Burnt lime	
				Sealed cured at 25 °C	19.6 MPa		
Cameroon VA	Paste	NS + NH (12 M)	Alumina (Al_2_O_3_)	Sealed 24 h. Ambient temperature (24 ± 3 °C)	47.8 MPA	40% Alumina	Tchakouté et al. [35]
Fused-Cameroon VA with NaOH	Mortar	NS	Metakaolin	Sealed 24 h. Ambient temperature (24 ± 3 °C)	68.8 MPA	60% Metakaolin	Tchakouté et al. [34]
Colombian VA	Paste	NS + NH SiO_2_/Al_2_O_3_ molar ratios 6.0–8.0 Na_2_O/SiO_2_ molar ratios 0.05–0.20	GBFS ^6^	Sealed 24 h. Humidity 90% at 25 °C until the test age	125 MPa	6.5–7.0 SiO_2_/Al_2_O_3_, 30% GBFS	Robayo-Salazar et al. [27]
Iran VA	Mortar	NS + NH Ms = 1, 1.5, 2 SiO_2_/Al_2_O_3_ = 2.35	CAC ^7^	24 h at 23.0 ± 2.0 °C and a RH > 95% hydrothermal conditions at 95 °C for 20 h	65 MPa	24% CAC	Vafaei &. Allahverdi [54]
Cameroon VA	Paste	NH (12 M) + NS (Ms = 1.1 & 1.4)	Metakaolin	Sealed 24 h. Ambient temperature (24 ± 3 °C)	69 MPa	25% Metakaolin	Djobo et al. [84]
Cameroon VA	Paste	NH + NS (SiO_2_/Na_2_O =1.6)	GBFS	Stored at 25 °C until the 3, 7 and 28-days compressive strength test.	85 MPa	50% Slag	Lemougna et al. [92]

^1^ Volcanic ash; ^2^ KOH; ^3^ Na_2_SiO_3_; ^4^ NaOH; ^5^ Modulus of silica; ^6^ Granulated blast furnace slag; ^7^ Calcium aluminate cement.

**Table 5:** Summary of representative studies on alkali-activated VA-based mortars.

Binder	Types of Activators/ Alkaline Solution		Aggregates (A)	Curing Conditions	Compressive Strength	Ref.
VA ^1^	NH ^2^, NS ^3^, Ms ^4^	S/B ^5^	A/B ^6^			
Volcanic Tuff < 45 µm, specific gravity = 2.38	NH (10, 12, 14, 16 M)	0.35 0.45	River aggregates (0–2, 2–4 and 4–8 mm), A/B = 2	Curing in covered molds at 90, 120, 150 °C for 72 h, then samples were kept in laboratory conditions until the test day.	25.83 MPa at 90 days	Kantarci et al. [55]
	NS (Ms = 0.6, 0.7, 0.8, 0.9, 1.0) + NH (10 M)			Curing in covered molds at 90, 105, 120 °C for 72 h then samples were kept in laboratory conditions until the test day.	37.09 MPa at 90 days	
VA < 200 µm, specific gravity = 2.62	NS + NH (12 M) Mass ratio of NS/NH = 2.4	0.45	Sand (specific gravity 2.55) A/B = 2	Room temperature (27 ± 3 °C) for 7-days 80 °C for 24 h The specimens after casting were demolded and keep at ambient temp until test performed.	37.9 MPa at 90 days	Djobo et al. [22]
VA, specific gravity = 2.29 SSA ^7^ = 6088 cm^2^/g	NH (2.5, 5, 7.5, 10, 12.5 M)	0.50, 0.54, 0.58	Fine aggregate (specific gravity = 2.76) A/B = 2.0	Pre-curing: 60 °C for 3 h in an oven before demolding Then oven at 80 °C (exposed, sealed and moist cured; 1, 3 and 7 days)	31.8 MPa at 7 days (exposed)	Ghafoori et al. [46]
					26.6 MPa at 7 days (wet)	
					37.7 MPa at 7 days (sealed)	
VA < 440 µm	NH Na_2_O/SiO_2_ * (0.15–0.30)	0.21	Sand (density 2.56 g/cm^3^) (40% by wt.)	Dry curing in the open air at 90 °C for 5 days	30 MPa at 28 days for a sample with Na_2_O/SiO_2_ molar ratio of 0.30	Lemougna et al. [36]

^1^ Volcanic ash; ^2^ NaOH; ^3^ Na_2_SiO_3_; ^4^ Modulus of silica; ^5^ Solution-to-binder ratios; ^6^ Aggregate-to-binder ratios; ^7^ Specific surface area. * Composition of the samples with five Na_2_O/SiO_2_ molar ratios synthetized from VA into a solution of NH in distilled water.

**Table 6:** Mixture proportions (kg/m^3^) for alkali-activated concretes developed in different investigations, as detailed in Table 7.

Research Author	VA ^1^ (kg/m^3^)	CA ^2^ (kg/m^3^)	FA ^3^ (kg/m^3^)	Silica Sand (kg/m^3^)	NS ^4^ (kg/m^3^)	AH ^5^ (kg/m^3^)	W ^6^ (kg/m^3^)	SP ^7^ (%)	w/b ^8^
Ibrahim et al. [57]	400	1206	650	–	150	60	100	–	0.25
Robayo-Salazar et al. [5]	400	933	763	–	146		103	–	0.35
Haddad et al. [58]	410	740	666	444	132	53	83	3	0.40
Bondar et al. [94]	391	1229	578	–	34 *	66	180	–	0.45
	344	1121	702	–	37 *	72	195	–	0.55
	417	1283	499	–	32 *	67	180	–	0.42
	417	1283	499	–	32 *	67	180	–	0.42

^1^ Volcanic ash; ^2^ Coarse aggregate; ^3^ Fine aggregate; ^4^ Na_2_SiO_3_; ^5^ Alkaline hydroxide (A= NH or KH); ^6^ Water; ^7^ Superplasticizer; ^8^ Water-to-binder ratios. * Values reported in cc/m^3^.

**Table 7:** Summary of studies that produced alkali-activated VA-based concretes.

Precursors VA ^1^ (SSA ^2^)		Alkaline Activators NS ^3^, NH ^4^, KH ^5^	Aggregates (FA ^6^, CA ^7^)	Curing Conditions	CS ^8^	Optimal Conditions	Ref.
VA (SSA, 442 m^2^/kg)		NS + NH(14 M) NS/NH = 2.0, 2.5, 2.75	FA: Dune Sand CA: Limestone	Oven at 60 °C for 1, 3, 7, 14 and 28 days	37.52 MPa at 7 days	NS/NH = 2.5 and 7 days of curing	Ibrahim et al. [57]
70% VA (SiO_2_/Al_2_O_3_, 6.79) + 30% GBFS (SiO_2_/Al_2_O_3_, 4.93)		NS + NH (SiO_2_/Na_2_O, 1.09)	FA: Sand CA: Gravel	Room temperature at 25 °C and RH > 80% until reaching their test age.	21 MPa at 28 days and 33 MPa at 360 days	NS/NH = 2.5 Room temperature at 25 °C and RH > 80%	Robayo-Salazar et al. [5]
VA (fineness 700 m^2^/kg, SiO_2_/Al_2_O_3_)		NS + NH(14 M) NS/NH = 0.5–3.0	FA: Mineral (SG, 2.53) CA: Limestone (SG, 2.44) SP ^9^: Polycarboxylate up to 6% by wt. of VA	Dry curing at room temperature for 7 and 28 days	24.1 MPa at 28 days	NS/NH = 2.5 Dry curing period for 24 h at 80 °C	Haddad & Alshbuol [58]
				Dry curing period at 40, 80 and 120 °C for 24 h and 48 h and kept in air for the rest of the 28 days until testing	31.9 MPa at 28 days		
VAs <75 µm:	Type 1 SSA, 3836 cm^2^/g	NS+ KH (7.5 M) KH/NS = 7.1	FA: sand CA: Gravel (SG, 2.6)	Sealed curing at 20, 40 and 60 ± 2 °C Fog curing at 20, 40 and 60 ± 2 °C	40.97 MPa at 180 days	Cured at 40 °C sealed	Bondar et al. [94]
	Type 2 SSA, 10621 cm^2^/g	KH/NS = 7.7			33.15 MPa at 90 days	Cured at 60 °C sealed	
	Type 3 (Calcined) SSA, 5500, cm^2^/g	KH/NS= 7.7			40.56 MPa at 180 days	Cured at 20 °C sealed	

^1^ Volcanic ash; ^2^ Specific surface area; ^3^ Na_2_SiO_3_; ^4^ NaOH; ^5^ KOH; ^6^ Fine aggregate; ^7^ Coarse aggregate; ^8^ CS: Compressive strength; ^9^ SP: Superplasticizer.

## Data Availability

Not applicable.
